# Supplementary material for: Prevalence and incidence of young onset dementia and associations with comorbidities: A study of data from the French national health data system
Source: PLoS Med. 2021 Sep 23;18(9):e1003801. doi: 10.1371/journal.pmed.1003801 (PMC8496799; doi:10.1371/journal.pmed.1003801)
Supplement: S1 Table — SNDS, Système National des Données de Santé. (DOCX) [file pmed.1003801.s002.docx]

S1 Table. Algorithms for the identification of comorbidities and treatments of interest in the SNDS.

|  |  | **Sources of information and depth in algorithms** | | |
| --- | --- | --- | --- | --- |
| **Conditions** | **List of ICD-10 codes** | **Hospital discharge diagnoses (ICD-10 codes)** | **LTD Diagnosis (ICD-10 codes)** | **Drug reimbursment claims^a^**  **(ATC codes)** |
| **Neurological diseases** |  |  |  |  |
| Parkinson’s disease | F02.3, G20 | Year t to t-4^b^ | Year t | Year t |
| Epilepsy | G40, G41 | Year t to t-4 | Year t | - |
| Multiple sclerosis | G35 | Year t to t-4 | Year t | - |
| **Psychiatric disorders and related drug therapies** |  |  |  |  |
| Substance abuse disorders (drugs, alcohol, cannabis) | F10 to F19 | Year t to t-1 | Year t | - |
| Schizophrenia and psychotic disorders | F20 to F25, F28 to F29 | Year t to t-4 | Year t | Year t |
| **Cardiovascular, cerebrovascular and metabolic diseases, and drug related therapy** | |  |  |  |
| Morbid obesity | E66 | Year t to t-4 |  |  |
| Diabetes | E10 to E14, G59.0, G63.2, G73.0, G99.0, H28.0, H36.0, I79.2, L97, M14.2, M14.6, N08.3 | Year t to t-1 | Year t | Year t to t-1 |
| Acute cerebrovascular disease (excluding transient attacks) | I60 to I64 | Year t | - | - |
| Sequelae of cerebrovascular disease of history of acute cerebrovascular disease | I60 to I64, I67 to I69, G81 | Year t to t-4 | Year t | - |
| Chronic ischemic heart disease or history of acute ischemic heart disease | I20 to I25 | Year t to t-4 | Year t | - |
| Acute ischemic heart disease | I21 to I24 | Year t | - |  |
| Cardiac arrhythmias and conduction disorders | I44, I45, I47 to I49 | Year t to t-4 | Year t |  |
| Antihypertensive drug therapy | - | - | - | Year t |
| **Traumatic brain injury** | F07.2, S02.0, S02.1, S02.3, S02.7, S02.8, S02.9, S06, S07.1, T90.2, T90.5 | Year t to t-4 |  |  |

SNDS, Système national des données de santé; ICD-10, international classification of diseases 10^th^ revision; LTD, long term disease; ATC, anatomical therapeutic chemical.

^a^ Detailed information regarding ATC codes are available in <https://www.ameli.fr/fileadmin/user_upload/documents/Methodologie_medicale_cartographie.pdf>.

^b^  t=2016 in this study, t-4=2012
