# Supplementary material for: Prevalence and incidence of young onset dementia and associations with comorbidities: A study of data from the French national health data system
Source: PLoS Med. 2021 Sep 23;18(9):e1003801. doi: 10.1371/journal.pmed.1003801 (PMC8496799; doi:10.1371/journal.pmed.1003801)
Supplement: S2 Table — PR, prevalence rate; YOD, young onset dementia. (DOCX) [file pmed.1003801.s003.docx]

S2 Table. Prevalence rates (per 100,000 persons) of YOD in France on December 31st, 2016.

| **Population** | **Age (years)** | **N YOD** | **Population^a^** | **Prevalence rate** | **95% CI** |
| --- | --- | --- | --- | --- | --- |
| ***Overall*** |  |  |  |  |  |
|  | 40-44 | 944 | 3,742,220 | 25.2 | (23.7; 26.9) |
|  | 45-49 | 1,553 | 3,778,531 | 41.1 | (39.1; 43.2) |
|  | 50-54 | 2,658 | 3,660,977 | 72.6 | (69.9; 75.4) |
|  | 55-59 | 4,635 | 3,287,289 | 141.0 | (137; 145.1) |
|  | 60-64 | 8,676 | 3,046,298 | 284.8 | (278.9; 290.9) |
|  | Total | 18,466 | 17,515,315 | 105.4 | (103.9; 107.0) |
| ***Men*** |  |  |  |  |  |
|  | 40-44 | 586 | 1,860,254 | 31.5 | (29.1; 34.2) |
|  | 45-49 | 896 | 1,860,544 | 48.2 | (45.1; 51.4) |
|  | 50-54 | 1,510 | 1,776,211 | 85.0 | (80.8; 89.4) |
|  | 55-59 | 2,520 | 1,550,210 | 162.6 | (156.3; 169.0) |
|  | 60-64 | 4,499 | 1,404,073 | 320.4 | (311.2; 329.9) |
|  | Total | 10,011 | 8,451,292 | 118.5 | (116.2; 120.8) |
| ***Women*** |  |  |  |  |  |
|  | 40-44 | 358 | 1,881,966 | 19.0 | (17.2; 21.1) |
|  | 45-49 | 657 | 1,917,987 | 34.3 | (31.7; 37.0) |
|  | 50-54 | 1,148 | 1,884,766 | 60.9 | (57.5; 64.5) |
|  | 55-59 | 2,115 | 1,737,079 | 121.8 | (116.7; 127.1) |
|  | 60-64 | 4,177 | 1,642,225 | 254.4 | (246.8; 262.2) |
|  | Total | 8,455 | 9,064,023 | 93.3 | (91.3; 95.3) |

^a^ Number of persons affiliated to the general scheme of the social security (data are provided by the French national health insurance register (RNIAM))( <https://assurance-maladie.ameli.fr/sites/default/files/2016_effectif-par-pathologie-classe-age-sexe_serie-annuelle.xls>).
