# Supplementary material for: Prevalence and incidence of young onset dementia and associations with comorbidities: A study of data from the French national health data system
Source: PLoS Med. 2021 Sep 23;18(9):e1003801. doi: 10.1371/journal.pmed.1003801 (PMC8496799; doi:10.1371/journal.pmed.1003801)
Supplement: S3 Table — IR, incidence rate; YOD, young onset dementia. (DOCX) [file pmed.1003801.s004.docx]

S3 Table. Incidence rates (per 100,000 person-years) of YOD in France in 2016.

| **Population** | **Age (years)** | **N YOD** | **Population^a^** | **Incidence rate** | **95% CI** |
| --- | --- | --- | --- | --- | --- |
| ***Overall*** |  |  |  |  |  |
|  | 40-44 | 225 | 3,776,930 | 6.0 | (5.2; 6.8) |
|  | 45-49 | 332 | 3,753,743 | 8.8 | (7.9; 9.8) |
|  | 50-54 | 616 | 3,622,905 | 17.0 | (15.7; 18.4) |
|  | 55-59 | 1,016 | 3,251,985 | 31.2 | (29.4; 33.2) |
|  | 60-64 | 1,886 | 3,023,837 | 62.3 | (59.6; 65.3) |
|  | Total | 4,074 | 17,429,399 | 23.4 | (22.7; 24.1) |
| ***Men*** |  |  |  |  |  |
|  | 40-44 | 138 | 1,874,622 | 7.4 | (6.2; 8.7) |
|  | 45-49 | 196 | 1,845,638 | 10.6 | (9.2; 12.2) |
|  | 50-54 | 343 | 1,753,558 | 19.6 | (17.6; 21.7) |
|  | 55-59 | 548 | 1,530,136 | 35.8 | (32.9; 38.9) |
|  | 60-64 | 1,031 | 1,394,833 | 73.8 | (69.5; 78.6) |
|  | Total | 2,256 | 8,398,786 | 26.9 | (25.8; 28.0) |
| ***Women*** |  |  |  |  |  |
|  | 40-44 | 87 | 1,902,308 | 4.6 | (3.7; 5.6) |
|  | 45-49 | 136 | 1,908,105 | 7.1 | (6.0; 8.4) |
|  | 50-54 | 273 | 1,869,347 | 14.6 | (13.0; 16.4) |
|  | 55-59 | 468 | 1,721,849 | 27.2 | (24.8; 29.8) |
|  | 60-64 | 854 | 1,629,004 | 52.5 | (49.0; 56.1) |
|  | Total | 1,818 | 9,030,613 | 20.1 | (19.2; 21.1) |

**^a^** Number of person-years affiliated to the general scheme of the social security in 2016 (data are provided by the French national health insurance register (RNIAM)), corrected by the estimated number of YOD cases in 2016.
