# Supplementary material for: Prevalence and incidence of young onset dementia and associations with comorbidities: A study of data from the French national health data system
Source: PLoS Med. 2021 Sep 23;18(9):e1003801. doi: 10.1371/journal.pmed.1003801 (PMC8496799; doi:10.1371/journal.pmed.1003801)
Supplement: S4 Table — YOD, young onset dementia. (DOCX) [file pmed.1003801.s005.docx]

S4 Table. Association between comorbidities and sex in persons without YOD from the French population in 2016 (40-64 years).

|  |  | **Men** | |  | **Women** | |  |  | | | | | |  | |  | | | | |
| --- | --- | --- | --- | --- | --- | --- | --- | --- | --- | --- | --- | --- | --- | --- | --- | --- | --- | --- | --- | --- |
|  |  | **n** | **%** |  | **n** | **%** |  | **Crude OR^a^** | | **95% CI** | | **p-value** | |  | | **Adjusted OR^a,b^** | **95% CI** | | **p-value** | |
| **Cardiovascular, cerebrovascular and metabolic diseases and drug related therapies** | |  |  |  |  |  |  |  | |  | |  | |  | |  |  | |  | |
| Morbid obesity | | 297,469 | (4.0) |  | 432,510 | (5.0) |  | 0.79 | | (0.79-0.80) | | <.001 | |  | | 0.80 | (0.79-0.80) | | <.001 | |
| Diabetes | | 590,146 | (7.9) |  | 478,046 | (5.5) |  | 1.48 | | (1.47-1.48) | | <.001 | |  | | 1.52 | (1.51-1.53) | | <.001 | |
| Acute cerebrovascular disease (excluding transient attacks) | | 12,821 | (0.2) |  | 7,702 | (0.1) |  | 1.94 | | (1.89-2.00) | | <.001 | |  | | 1.97 | (1.91-2.03) | | <.001 | |
| Sequelae of cerebrovascular disease or history of acute cerebrovascular disease | | 83,211 | (1.1) |  | 65,238 | (0.7) |  | 1.49 | | (1.48-1.51) | | <.001 | |  | | 1.52 | (1.50-1.53) | | <.001 | |
| Acute ischemic heart disease | | 21,251 | (0.3) |  | 5,877 | (0.1) |  | 4.22 | | (4.10-4.35) | | <.001 | |  | | 4.28 | (4.16-4.40) | | <.001 | |
| Chronic ischemic heart disease or history of acute ischemic heart disease | | 294,471 | (3.9) |  | 86,571 | (1.0) |  | 4.09 | | (4.05-4.12) | | <.001 | |  | | 4.25 | (4.22-4.29) | | <.001 | |
| Cardiac arrhythmias and conduction disorders | | 115,950 | (1.5) |  | 65,754 | (0.8) |  | 2.07 | | (20.5-2.09) | | <.001 | |  | | 2.12 | (2.10-2.14) | | <.001 | |
| Antihypertensive drug therapy | | 1,609,361 | (21.5) |  | 1,667,208 | (19.1) |  | 1.16 | | (1.15-1.17) | | <.001 | |  | | 0.832 | (0.830-0.834) | | <.001 | |
| **Neurological diseases other than dementia** | |  |  |  |  |  |  |  | |  | |  | |  | |  |  | |  | |
| Parkinson’s disease | | 13,249 | (0.2) |  | 13,326 | (0.2) |  | 1.16 | | (1.13-1.19) | | <.001 | |  | | 1.19 | (1.16-1.21) | | <.001 | |
| Epilepsy | | 52,804 | (0.7) |  | 40,526 | (0.5) |  | 1.52 | | (1.50-1.54) | | <.001 | |  | | 1.53 | (1.51-1.55) | | <.001 | |
| Multiple sclerosis | | 13,225 | (0.2) |  | 35,945 | (0.4) |  | 0.43 | | (0.42-0.44) | | <.001 | |  | | 0.43 | (0.42-0.44) | | <.001 | |
| **Psychiatric disorders and related drug therapies** | |  |  |  |  |  |  |  | |  | |  | |  | |  |  | |  | |
| Substance abuse disorders (drugs, alcohol, cannabis) | | 106,667 | (1.4) |  | 47,748 | (0.5) |  | 2.63 | | (2.59-2.66) | | <.001 | |  | | 2.62 | (2.59-2.65) | | <.001 | |
| Schizophrenia and psychotic disorders | | 114,518 | (1.5) |  | 90,203 | (1.0) |  | 1.49 | | (1.47-1.50) | | <.001 | |  | | 1.49 | (1.47-1.50) | | <.001 | |
| **Traumatic brain injury** | | 45,224 | (0.6) |  | 20,909 | (0.2) |  | 2.53 | | (2.49-2.57) | | <.001 | |  | | 2.45 | (2.50-2.58) | | <.001 | |
| Young onset dementia, YOD; | | | | | | | | |  | |  | |  | |  | |  |  | |  |
| **^a^** Odds ratios (OR), 95% confidence intervals (CI), and p-values were computed using logistic regression. | | | | | | | | |  | |  | |  | |  | |  |  | |  |
| **^b^** OR adjusted for age and sex. | |  |  |  |  |  |  |  | |  | |  | |  | |  |  | |  | |
