# Supplementary material for: Prevalence and incidence of young onset dementia and associations with comorbidities: A study of data from the French national health data system
Source: PLoS Med. 2021 Sep 23;18(9):e1003801. doi: 10.1371/journal.pmed.1003801 (PMC8496799; doi:10.1371/journal.pmed.1003801)
Supplement: Attachment — Submitted filename: Point by point response.docx [file pmed.1003801.s006.docx]

**Point by point response to reviewers and editor’s comments**

**Requests from the editors:**

Title: Please revise your title according to PLOS Medicine's style. Your title must be nondeclarative and not a question. It should begin with main concept if possible. "Effect of" should be used only if causality can be inferred, i.e., for an RCT. Please place the study design ("A randomized controlled trial," "A retrospective study," "A modelling study," etc.) in the subtitle (ie, after a colon).

🡺 We revised the title according to your recommandation: “Young onset dementia: prevalence, incidence, and role of comorbidities using French administrative databases”.

2. Data Availability Statement: The Data Availability Statement (DAS) requires revision. For each data source used in your study:

a) If the data are freely or publicly available, note this and state the location of the data: within the paper, in Supporting Information files, or in a public repository (include the DOI or accession number).

b) If the data are owned by a third party but freely available upon request, please note this and state the owner of the data set and contact information for data requests (web or email address). Note that a study author cannot be the contact person for the data.

c) If the data are not freely available, please describe briefly the ethical, legal, or contractual restriction that prevents you from sharing it. Please also include an appropriate contact (web or email address) for inquiries (again, this cannot be a study author).

🡺 We revised this section.

3. Line numbers: Please provide line numbers throughout the manuscript with the revised version.

🡺 We added line numbers.

4. Author Summary: Please move the Author Summary so that it follows the Abstract. Please format using bullet points, with 2-3 points for each section. This text is subject to editorial change and should be distinct from the scientific abstract. Please see our author guidelines for more information: <https://journals.plos.org/plosmedicine/s/revising-your-manuscript#loc-author-summary>

🡺 We revised this section according to the guidelines.

5. Abstract: Methods and Findings: Please provide some more detail on the study population. Please quantify the main results (with 95% CIs and p values) and please include the important dependent variables that are adjusted for in the analyses. * In the last sentence of the Abstract Methods and Findings section, please describe the main limitation(s) of the study's methodology.

🡺 We revised this section.

6. Abstract: Conclusions: * Please address the study implications without overreaching what can be concluded from the data; the phrase "In this study, we observed ..." may be useful.

🡺 We revised this section.

7. In-text citations: Please use numbers within square brackets for in-text citations. Where more than one reference is indicated, please do not include spaces within brackets.

🡺 We formatted the references as recommended.

8. Methods: Please ensure that the study is reported according to the STROBE guideline, and include the completed STROBE checklist as Supporting Information. Please add the following statement, or similar, to the Methods: "This study is reported as per the Strengthening the Reporting of Observational Studies in Epidemiology (STROBE) guideline (S1 Checklist)."

The STROBE guideline can be found here: <http://www.equator-network.org/reporting-guidelines/strobe/>

When completing the checklist, please use section and paragraph numbers, rather than page numbers.

🡺 We now provide the STROBE checklist as a supplementary file and added the required statement to the methods (line 180)

9. Methods: Prospective Analysis Plan: Did your study have a prospective protocol or analysis plan? Please state this (either way) early in the Methods section.

a) If a prospective analysis plan (from your funding proposal, IRB or other ethics committee submission, study protocol, or other planning document written before analyzing the data) was used in designing the study, please include the relevant prospectively written document with your revised manuscript as a Supporting Information file to be published alongside your study, and cite it in the Methods section. A legend for this file should be included at the end of your manuscript.

b) If no such document exists, please make sure that the Methods section transparently describes when analyses were planned, and when/why any data-driven changes to analyses took place.

c) In either case, changes in the analysis-- including those made in response to peer review comments-- should be identified as such in the Methods section of the paper, with rationale.

🡺This work was performed as part of the surveillance of neurodegenerative diseases in France by Santé publique France, the French surveillance government agency. This project is part of a larger surveillance program. No IRB or ethics committee were necessary as the work was performed using administrative databases to which Santé publique France has permanent access.

🡺 A sentence from the introduction (lines 96) explains why we performed this specific study following preliminary work. We feel that this sentence fits well in the introduction; we would be happy to move it to the methods section if you prefer, we then would change the end of the introduction: *« In France, a preliminary study compared dementia prevalence based on administrative data to those from cohort studies, and showed that administrative data underestimated prevalence after 70 years but yielded consistent estimates for YOD.[27] We hypothesized that the dramatic impact of YOD on patient’s lives is more likely to lead younger individuals to be in contact with the health system than older subjects making administrative data relevant to study YOD epidemiology. »*

🡺 In addition, analysis of the role of comorbidites on sex-difference resulted from our finding of a difference in YOD rates between men and women.We added a sentence in the statistical analysis paragraph of the methods regarding this part of the study (lines 176): *« As we found a strong difference in YOD incidence by sex, we further evaluated the contribution of comorbidities to the sex-differences in YOD incidence by estimating the percentage reduction in the association between sex and YOD after adjustment for comorbidities. »*

10. Results: Please provide p values and 95% confidence intervals for all statistical comparisons, and please present both unadjusted and adjusted results (at least in tables, if not in the text.) Where p values are presented, please clarify the statistical tests used.

🡺 We revised all tables and added p-values for all statistical comparisons where appropriate. In addition, we included unadjusted and adjusted odds ratios in table 2 and supplementary table 4.

11. Discussion: First sentence: Please avoid claims of primacy, or if necessary, please temper with “to the best of our knowledge” or similar.

🡺 We revised this sentence and the rest of the manuscript in light of this comment.

12. Discussion: If relevant for sex/gender differences, please consider discussing head trauma/ injury, which could be associated with motor vehicle accidents or sports related and may be more prominent in men, and this seems like it could potentially contribute to differences as a risk factor for cognitive decline and dementia.

🡺 Thank you very much for this important comment which led us to perform additional analyses regarding the role of TBI and its association with YOD. As anticipated by the editor, we found that TBI was an important contributor to the sex difference in YOD incidence. The methods, results, and discussion section as well as the tables have been modified in order to include this point.

13. Discussion: Please present and organize the Discussion as follows: a short, clear summary of the article's findings; what the study adds to existing research and where and why the results may differ from previous research; strengths and limitations of the study; implications and next steps for research, clinical practice, and/or public policy; one-paragraph conclusion.

🡺 We revised the discussion and added subtitles to clarify its structure.

14. References: Please use the "Vancouver" style for reference formatting, and see our website for other reference guidelines <https://journals.plos.org/plosmedicine/s/submission-guidelines#loc-references>

🡺 The format of the references has been modified.

15. Table 2 and Table 3: Please provide both adjusted and unadjusted analyses.

🡺 We now provide unadjusted OR in table 2. Regarding table 3, unadjusted analyses are not appropriate, as our aim is to show the percentage reduction of the sex effect after adjusting for each other covariable. Hence, OR are necessiraly adjusted.

16. Supplementary Table 1: Please define all abbreviations in the legend.

🡺 Done.

17. Supplementary Table 4: Please provide both the adjusted and unadjusted analyses.

🡺 We now provide both adjusted and unadjusted OR in supplementary table 4.

**Comments from the reviewers:**

**Reviewer #1**: Using the French national health data system, this study attempts to estimate YOD prevalence and incidence in 2016 of French individuals aged 40-64y and study the contribution of comorbidities to YOD incidence.

1- 2016 is five years ago now. Can the analysis be updated so it is more relevant to todays prevalence and incidence rates?

🡺 We wish we could provide more recent data for this analysis. Unfortunately, our algorithm to detect YOD cases is based on three criteria among which the use of anti-Alzheimer’s medication. In France, in 2017, a new governemental decision was made to stop reimbursing anti-Alzheimer’s medication following the advice delivered by the High Authority for Health in 2016 and 2018 stating an "insufficient medical benefit and dangerousness because of significant side effects"(Krolak-Salmon, P., et al. (2018). "France Will No More Reimburse Available Symptomatic Drugs Against Alzheimer's Disease." J Alzheimers Dis 66(2): 425-427).

As a consequence and as shown in the figure below, the number of subjects <65y with anti-Alzheimer’s reimbursements in our database declined sharply after 2016 and almost no patients used anti-Alzheimer’s medications in 2019. This implies that studies using more recent data would miss a significant number of patients who are not treated anymore and would lead to underestimate prevalence and incidence rates of dementia. This issue coud also lead to selection bias, because patients with lower socio-economic level who cannot afford to pay the drugs would be more likely to be missed than patients with higher socio-economic level.

In addition, incidence rates are unlikely to have dramatically changed over 5 years. Therefore, the conclusions from our study are likely to be still relevant in 2021.

|  | Number of subjects with at least 2 anti-alzheimer's reimbursment |
| --- | --- |
| 2014 | 3891 |
| 2015 | 3718 |
| 2016 | 3501 |
| 2017 | 3162 |
| 2018 | 2579 |
| 2019 | 83 |

Figure : Number of subjects (40-64 years) with at least 2 anti-Alzheimer’s reimbursments in one year between 2014 and 2019

2- "Based on the LOD literature, we selected cardio- and cerebro-vascular diseases and treatments, metabolic conditions, psychiatric diseases, psychotropic drugs, and other neurological diseases that may be YOD risk factors."

Could the authors please provide citations for reference here?

🡺 Thank you for this comment. We have added additional references in the text (methods, paragraph « comorbidities assesment », lines 142). Please note that following a comment from the editor, we have now added traumatic brain injury (TBI) to the list of covariates.

*“we selected cardio- and cerebro-vascular diseases and treatments, metabolic conditions, psychiatric diseases, psychotropic drugs, other neurological diseases, and traumatic brain injury as a potential risk factors for YOD.[29-35]”*

29.	Aarsland D, Kurz MW. The epidemiology of dementia associated with Parkinson's disease. Brain pathology. 2010;20(3):633-9. doi: 10.1111/j.1750-3639.2009.00369.x. PMID: 20522088.

30.	Chen Y, Sillaire AR, Dallongeville J, Skrobala E, Wallon D, Dubois B, et al. Low Prevalence and Clinical Effect of Vascular Risk Factors in Early-Onset Alzheimer's Disease. J Alzheimers Dis. 2017;60(3):1045-54. Epub 2017/10/07. doi: 10.3233/jad-170367. PMID: 28984595.

31.	Deckers K, Schievink SHJ, Rodriquez MMF, van Oostenbrugge RJ, van Boxtel MPJ, Verhey FRJ, et al. Coronary heart disease and risk for cognitive impairment or dementia: Systematic review and meta-analysis. PLoS One. 2017;12(9):e0184244. Epub 2017/09/09. doi: 10.1371/journal.pone.0184244. PMID: 28886155.

32.	Gardner RC, Yaffe K. Epidemiology of mild traumatic brain injury and neurodegenerative disease. Mol Cell Neurosci. 2015;66(Pt B):75-80. Epub 2015/03/10. doi: 10.1016/j.mcn.2015.03.001. PMID: 25748121.

33.	Gladman JT, Corriveau RA, Debette S, Dichgans M, Greenberg SM, Sachdev PS, et al. Vascular contributions to cognitive impairment and dementia: Research consortia that focus on etiology and treatable targets to lessen the burden of dementia worldwide. Alzheimers Dement (N Y). 2019;5:789-96. doi: 10.1016/j.trci.2019.09.017. PMID: 31921967.

34.	Ribe AR, Laursen TM, Charles M, Katon W, Fenger-Gron M, Davydow D, et al. Long-term Risk of Dementia in Persons With Schizophrenia: A Danish Population-Based Cohort Study. JAMA Psychiatry. 2015;72(11):1095-101. Epub 2015/10/08. doi: 10.1001/jamapsychiatry.2015.1546. PMID: 26444987.

35.	Zelano J, Brigo F, Garcia-Patek S. Increased risk of epilepsy in patients registered in the Swedish Dementia Registry. Eur J Neurol. 2019. Epub 2019/07/23. doi: 10.1111/ene.14043. PMID: 31330091.

3- The application of Poisson regression to estimate 95% confidence intervals (CI), and male-to-female prevalence (PRRM/W) and incidence (IRRM/W) rate ratios and 95% CI, overall (adjusted for age) and by age, as well as including an interaction term between age and sex, is a technically appropriate and robust methodological approach.

🡺 Thank you for this positive comment.

4- "The IRRM/W was 1.61 (95% CI=1.23-2.11) between 40-44 years and it progressively decreased to 1.41 (95% CI=1.29-1.54) between 60-64 years. " These results show overlapping CIs, that suggest that in fact the difference is not statistically significant? Did the authors consider undertaking any trend analyses?

🡺 Thank you for this comment. We performed a trend analyses to test whether the sex ratio decreased with increasing age (line 166): *« We tested whether PRRM/W and IRRM/W changed with age by including an interaction term between continuous age and sex ».*

🡺 The interaction between continuous age and sex was significant for the PRR_M/W_ (p<0.0001) but not for the IRR_M/W_ (p=0.494). However, please note that the age-specific IRR_M/W_ are similar to the corresponding age-specific PRR_M/W_ and follow the same general decreasing trend with increasing age; the non-significant interaction for IRR_M/W_ and overlapping confidence intervals are likely due to insufficient statistical power to detect a significant trend as the number of incident cases in each age group is considerably smaller than the number of prevalent cases.

We included the following sentence in the results section (line 207): *« PRR_M/W_ decreased with age (Figure 2). It was 1.66 (95% CI=1.45-1.89) between 40-44 years and it progressively decreased to 1.26 (95% CI=1.21-1.31) between 60-64 years (p-interaction between sex and continuous age < 0.0001). Age-specific IRR_M/W_ were similar to corresponding age-specific PRR_M/W_, and there was a similar trend of decreasing IRRM/W with continuous age but it was not statistically significant (p-interaction=0.494), likely due to insufficient statistical power as the number of incident cases in each age group was considerably smaller than the number of prevalent cases. »*

5- Can the authors please present the uncertainty (i.e. the confidence intervals, as shown in supplementary tables 2 and 3) in the PR and IR's in Figure 1?

🡺 We added confidence intervals to Figure 1.

6- Can the authors please complete statistical testing of differences between groups in Table 1?

🡺 We compared subjects’characteristics by sex in table 1. We added this point in the methods section (line 153): *“Characteristics of subjects with prevalent and incident YOD overall and by sex are presented using means (standard deviation) and numbers (proportions). Differences between sexes were tested using student t-test and Pearson’s chi-square for continuous and categorical variables, respectively.”*

🡺Please note that due to the large sample size of prevalent cases, differences may be statistically significant although they are not large.

7- Table 2: Can the authors please include parentheses for the percentage figures?

🡺 We included parentheses for the percentage figures in table 2 and supplementary table 4

**Reviewer #2:** The manuscript of Carcaillon-Bentata et al provides YOD prevalence and incidence estimates in France and the contribution of comorbidities to YOD incidence. To this aim, the authors used the French national health data system. The results they found were they were around 24.000 YOD cases in France in 2016. Further, cardio and cerebrovascular, neurological, and psychiatric disease prevalence explained more than 50% of the sex difference in YOD incidence. The authors highlight that this result supports the idea of preventive approaches aimed at reducing or postponing the onset of dementia by acting on cardiovascular risk factors in mid-life.

1- This manuscript has strengths that should be noted. First, the use of the French national health data system which covers more than 98% of the French population. Second, the well-written manuscript. However, I have some doubts about the relevance and the interpretation of the results.

🡺 Thank you for this summary and the positive comments.

2- My first main concern is regarding the interpretation of the findings. The authors have only explored the incidence of YOD encompassing the different types of dementia. It is well-known that the incidence of different types of dementia differs. Due to the higher sample, I would strongly suggest doing a breakdown of the prevalence of different dementia subgroups showing the results in a table with the frequency of clinical diagnosis among EAD cases and incidence rate per 100.000 person-years. Knowing the difference between subgroups would allow for a more powerful discussion of the findings. Also, the impact of comorbidities depends on the type of dementia. Furthermore, previous literature has shown the sex differences in some kinds of dementia, such as Alzheimer's Disease (Ferretti, et al. 2020) and Behavioural Variant of Frontotemporal Dementia (Illán-Gala, et al. 2021). However, the sex difference in the incidence of subtypes of YOD is lacking and deserves further study.

Therefore, authors should consider adding this analysis to the current manuscript.

🡺 We fully agree with you that YOD incidence and prevalence rates by type of dementia would be of great interest as well as studying sex-differences and the role of comorbidities in YOD subtypes.

Unfortunately, the administrative databases that we used for this study do not allow determining dementia subtypes accurately because there is no reliable information on the cause of dementia. One of the reasons may be that there may be an important variability in how ICD-10^th^ codes are attributed in different hospitals and by different doctors all over France. Among the three criteria used to identify dementia, LTD registrations and hospitalizations have associated ICD-10th codes that could be used for this purpose*.* However, we have observed that dementia ICD-10th codes often vary over time for the same subject. In addition, anti-Alzheimer drugs are also used for other diseases, such as PD with dementia. Further investigations and validation studies would be necessary before being able to use these codes to provide data on YOD subtypes.

We aknowledge that this is a major limitation of our study and emphasized this point in the limitations paragraph of the discussion (lines 331): *« Another important limitation pertains to the lack of accurate information regarding dementia subtypes; one of the reasons may be variability in how ICD-10^th^ codes are attributed in different hospitals and by different doctors all over France. Among the three criteria used to identify dementia, LTD registrations and hospitalizations have associated ICD-10th codes that could be used for this purpose. However, the accuracy of these codes and their relevance to identify dementia subtypes remain unclear, and additional validation studies are needed before they can be used for this aim.»*

3- Following the sex difference topic, in the current manuscript is not clear if the analysis of sex differences in YOD is one of the main aims or a secondary one. In my opinion, I would say that it is one of the main aims because in the methods part the authors specify that "we want to assess the contribution of comorbidities to sex-differences in EAD incidence". If this is correct, I was wondering why they analyzed it, have the authors a previous hypothesis? The manuscript should be rewritten focusing on this topic and giving it more prominence. For instance, in the introduction, there is no information about sex differences in YOD found in the previous literature. Besides, sex differences should be included as a keyword. However, I would strongly encourage the authors to keep this focused on the current manuscript. Elucidating sex differences in dementia, also in its incidence, will allow to development of a precision medicine approach encompassing sex-sensitive strategies

for prevention and detection.

🡺 Data regarding YOD frequency are scarce and missing in France. The primary objective of our study was to estimate YOD prevalence and incidence and to study the association between comorbidities and YOD incidence. When estimating prevalence and incidence rates, it is common to estimate age- and sex-specific rates, and this is mentioned in the last paragraph of the introduction stating the objective of our study (line 102): “*In this paper, we used more recent data from the French national health data system to estimate YOD prevalence and incidence in 2016, overall and by age and sex*”.

Analyses by sex are warranted because there is an important literature on sex-differences in dementia, and whether there are sex-differences for YOD is not well known; this issue is addressed in the discussion (lines 279). Because we found a strong sex-difference in YOD prevalence and incidence, we examined the role of comorbidities in sex-differences in YOD incidence. We added a sentence in the statistical analysis paragraph of the methods section to specify that this latter analysis was driven by results observed during the study (line 176): *« As we found a strong difference in YOD incidence by sex, we further evaluated the contribution of comorbidities to the sex-differences in YOD incidence by estimating the percentage reduction in the association between sex and YOD after adjustment for comorbidities. »*

4- It is well-known the difference between the concepts of sex and gender, referring to biology and social construct respectively. In this line, I was wondering if the gender factors can have an impact on the incidence of EAD as well.

🡺 We believe that gender is likely to have an impact on the incidence of YOD due to different behaviors in men and women. In particular, addictions and head injuries were more frequent in men than women, and these factors were strongly associated with YOD. Occupational exposures, which are also different between men and women, could also have an important impact on YOD.

This issue is mentioned in the discussion in the paragraph regarding sex differences (lines 291): *« Differences between men and women in dementia rates are likely to arise both from differences related to sex (i.e., biological) and gender (i.e., behaviours, such as additions; socio-cultural, such as education or occupation). »*

**Reviewer #3**: This paper addresses an important topic. Good prevalence and incidence rates for young onset dementia have been lacking and it is important to know these for purposes of policy, service provision and research. This paper therefore makes a welcome contribution to the literature. The report is clear and succinct. The discussion provides a very good comparison with others' findings and the conclusions make a valuable contribution to knowledge in this area.

🡺 Thank you for the positive comments.

I have only four points for consideration:

1.	In terms of your data source, I notice you accessed an impressive level (76% of the population) but this excludes those in public service. I wondered whether or not there is any systematic bias here that you could comment upon.

🡺 Indeed our selection excluded workers in public service (11%) but also farmers and agricultural workers and their spouses if unemployed (5%) as well as self-employed workers (6%), and other smaller health systems (2%).

We added this point to the Data sources in the methods section (line 122) : *“We excluded from our analyses workers in public services (11%), farmers, agricultural workers and their spouses if unemployed (5%), self-employed workers (6%), and workers from other smaller health systems (2%).”*

🡺 The subjects excluded from our study are very heterogenous. Public services concentrate a higher number of women and well educated individuals while agricultural and self-employed workers are two groups constituted of more men and with a large heterogeneity in terms of educational level. Therefore, and given the low amount of subjects that are missing, it is unlikely that the selection lead a systematic biais in our analysis.

We further added this sentence in the limitations section of the discussion (line 343).

2.	You did not refer to other comparable studies and their methods, strengths and limitations in the introduction, as might be conventional, but left this to the discussion. I think it would help to set the context to refer to these, at least briefly, in the introduction.

🡺 Thank you for this comment. We now provide more details regarding results from comparable studies in the introduction (lines 85) : *« In the context of population ageing, data on YOD is of major importance to anticipate needs for planning and allocation of health and social resources.[8] Some field and register studies provided heterogeneous estimates of YOD prevalence, ranging from 38 to 420/100,000 between 30 to 64 years [9-17]. Fewer studies are available regarding YOD incidence and methodological differences likely explain inconsistent results between them [9-11, 16-19]. In France, to our knowledge, only one study reported on the prevalence of young onset Alzheimer’s disease and found it to be 41.2/100,000 before the age of 60 years in 1998.[20]»*

3.	I am not a statistician so cannot comment on details of the analysis; however, as a lay person in this respect, I understood the expression, except for the correction used in calculating the incidence rate. This will be only my ignorance but, if shared with other readers, it may be worth considering how to make this more readily understandable.

🡺 Thank you for this comment, we agree that the sentence was not clear. Briefly, to estimate an incidence rate, the number of incident cases is divided by the number of persons at risk over a time period (expressed as person-years). Therefore, prevalent cases, who already have dementia, should be substracted from the numbers of persons at risk.

We simplified the sentence to clarify this issue (Methods, lines 160): *« The YOD incidence rate (IR) in 2016 was estimated by dividing the number of persons with incident dementia in 2016 by the number of person-years affiliated to the general scheme in 2016, from which we subtracted the number of persons with prevalent YOD in 2016.».*

4.	At the very end of the discussion section, you state that institutionalisation is [only?] available to those >60 years. This seems strange - may I check that this actually correct?

🡺 In the first version of the paper, we used the term institutionalisation instead of nursing home, which may lead to a misunderstanding. We now use the term “nursing home”, and we hope that this will be clearer. In France, only persons 60 years or older have access to nursing homes.

5.	I have two small suggestions in relation to writing style/terminology.

*	Terms used to describe young onset dementia vary but 'young onset dementia' has become the most common, superseding 'early onset dementia', since the latter can become confused with 'early-stage dementia'. I would suggest that you change your terminology to young onset dementia to ensure best fit with emerging nomenclature.

🡺 Thank you for this recommendation. We modified early onset dementia by young onset dementia in the whole manuscript

*	People living with dementia are very keen to be recognised and respected as whole human beings, especially as the condition is often equated with loss of personhood. In line with this, I would strongly urge you to consider replacing the term 'patient', where possible, with alternatives, e.g. in the first line of the abstract, you could say 'Dementia onset in those aged < 65 years….' And in the first paragraph of the background, you could say '…as the number of people affected….'. This helps to shift attitudes.

🡺 Thank you very much for these recommendations. We have made these changes throughout the paper.

**Reviewer #4**: This a well written, much needed study on the epidemiology of early onset dementia (YOD), a condition with dramatic consequences for the individual patients, their families and the society. It will be important to raise awareness about this condition and for improving planning and allocation of health and social resources. I strongly recommend its publication but I would advise to address the following comments to further improve it:

🡺 Thank you for the positive comments.

1)	It should made clear since the beginning, i.e. in the title and in the abstract, the fact that the epidemiological data on YOD where established from a register covering about 76% of the population of France. The current title and abstract give the uncorrect impression that the authors analysed data from the entire population of France.

🡺 Thank you for this comment. We have added in the abstract, author summary, and last sentence of the introduction the fact that used data for 76% of the French population (“Using data from the French national health data system for 76% of the French population, …”). It seems difficult to add this figure to the title, but to try to avoid any ambiguity, we have modified the title: we deleted “in France” and replaced “national” by “French” before “administrative databases”. The title now reads: “Young onset dementia: prevalence, incidence, and role of comorbidities using French administrative databases”.

2)	One major problem of the manuscript in its current version is the scarcity of references to previous epidemiological studies on early onset dementia, some of which are very recent (see as an example Chiari et al 2020). Sentences such as "Few studies have provided precise frequency estimates of YOD" (abstract) and "Some YOD frequency estimates come from registries but they are probably biased due to the inclusion of small numbers of selected patients referred to specialized centers" (introduction) need to be either played down or become more specific about what is actually not precise or lacking in recent work on the epidemiology YOD.

🡺 Thank you for this comment. We have revised the more recent literature and updated the reference list with three recent papers that are now mentioned in the introduction and discussion.

Awata, S., et al. (2020). "Prevalence and subtype distribution of early-onset dementia in Japan." Psychogeriatrics **20**(6): 817-823.

Chiari, A., et al. (2021). "Epidemiology of early onset dementia and its clinical presentations in the province of Modena, Italy." Alzheimers Dement **17**(1): 81-88.

Kvello-Alme, M., et al. (2020). "Incidence of Young Onset Dementia in Central Norway: A Population-Based Study." J Alzheimers Dis **75**(3): 697-704.

We also downplayed comments that could have been perceived as negative.

3)	The authors identified dementia cases among all individuals aged 40-64 years old. However, YOD can also occur before the age of 40. It would be very helpful if the authors could extend their analysis to include people aged 30-64. This will be more informative while also allowing a comparison with previous registry-based studies on the epidemiology of YOD (such as Garre-Olmo et al 2010). Please do specify in the first sentence of the Discussion that PR and IR are given for population aged 40-64 (again, I strongly suggest that you also add data regarding the 30-64 age group)

🡺When we planned our study, we made the choice to study YOD in subjects older than 40 years as the literature shows that dementia before this age is extremely rare. Unfortunately, we cannot consider adding data for those aged 30 to 40 years at this stage as we are working on a data extraction from our national health data system. We would need to start all over again the process to do a new selection which is not possible at this stage anymore.

🡺 We now added to the first sentence of the discussion (line 227) that our analyses are based on individuals aged 40 to 64 years old: *“To the best of our knowledge, we provide for the first time robust YOD PR and IR estimates in people aged 40 to 64 years-old in France”*

4)	One of the strategies for cases identification was the presence of reimbursements over a one-year period of anti-dementia drugs. Please note that acetylcholinesterase inhibitors and memantine are not generically anti-dementia but are specifically licensed for Alzheimer's disease. Although Alzheimer's disease is the most frequent cause of dementia, there are several other diseases causing YOD that especially present in young patients, such as frontotemporal dementia. Please change 'anti-dementia' to a more specific terminology and mention in the discussion that the inclusion of such a strategy may have biased case identification towards AD, possibly missing other dementias that do not have specific medications.

🡺 Than you for this comment. We have replaced anti-dementia by anti-Alzheimer’s drugs throughout the paper.

We agree that using anti-Alzheimer’s drugs may have shifted case-identification towars Alzheimer’s disease. However, please note that only 8% of the subjects that we identified were identified by anti-Alzheimer’s medications alone, and that these medications are being used, at least in France, for other causes of dementia, such as Parkinson’s disease, dementia with Lewy bodies, or mixed dementia.

We now mention this issue in the limitations paragraph of the discussion (lines 337): *« In addition, the use of anti-Alzheimer’s medications to identify YOD could have shifted our selection towards the inclusion of a higher number of persons with Alzheimer’s disease compared to other of dementia subtypes. However, the majority of persons with YOD were identified through LTD registration or hospitalizations, and only 8% by anti-Alzheimer’s medications alone. In addition, in France, some of the anti-Alzheimer’s medications can also be prescribed for other causes of dementia, such as Parkinson’s disease, dementia with Lewy bodies, or mixed dementia.»*

5)	In the discussion, the sentence "Field studies provided inconsistent results regarding YOD" needs to be re-framed. Actually, it may be argued that this sentence is outdated, as the only reference provided is a review paper of 2014, which includes studies published before 2014. Please add and discuss recently published original studies.

Similarly, the sentence "these estimates were generally based on few cases and imprecise" should be reframed. Please only consider stating that others' work is imprecise unless you have clear evidence and can prove with exact numbers such a strong judgment.

🡺 Following this comment and comment 2, we updated our reference list and took into account these new data in the introduction and discussion. We also downplayed comments that could have been perceived as negative. We used imprecise in a statistical sense, ie, with large confidence intervals, and did not mean to sound critical, but we understand the reviewer’s comments and deleted these statements.

6)	In the discussion please clearly consider that the lack of information about the type/etiology of dementia is a major limitation of the study, as this may be a much important limitation than the other limitations listed in the same sentence or at least equally important to the lack of information regarding dementia severity. Related to this please mention that the study is likely to be biased towards Alzheimer's disease, given that it is the only type of dementia that has specific medications (as for point 4)

🡺 We agree with the reviewer that the lack of information on dementia subtypes is an important limitation. Unfortunately, the administrative databases that we used for this study do not allow to determine the subtype of dementia accurately because there is no reliable information on the cause of dementia (please see our response to comment 2 from reviewer #2).

This issue is now discussed in more details in the limitations section of the discussion (lines 331). *« Another important limitation pertains to the lack of accurate information regarding dementia subtypes; one of the reasons may be variability in how ICD-10th codes are attributed in different hospitals and by different doctors all over France. Among the three criteria used to identify dementia, LTD registrations and hospitalizations have associated ICD-10th codes that could be used for this purpose. However, the accuracy of these codes and their relevance to identify dementia subtypes remain unclear, and additional validation studies are needed before they can be used for this aim. »*

We have also included a sentence about anti-Alzheimer’s medication (lines 334; please see our response to comment 4).

Minor comments:

The sentence "After 60 years, 6-9% of all dementia cases occurred before 65 years" in the first paragraph of the introduction is not easily readable. Doo the authors mean "Among the people older than 60,. …"

🡺 Thank you very much, we have modified this sentence as suggested.

What is the reference for the study mentioned in "one from Tuscany (Italy, 50-70 years: PR=100/100,000; IR=30/100,000 person-years)"?

🡺 These figures are extracted from the paper from Perera et al. who used databases from 5 countries. We agree that our sentence was misleading and have rephrased it (lines 253).

*“Recently, the European medical information framework consortium analyzed six electronic health databases from five countries to estimate dementia prevalence and incidence rates.[44] Our results are comparable to those from two databases identifying dementia using general pharmacy and hospital records: one from Tuscany (Italy, 50-70 years: PR=100/100,000; IR=30/100,000 person-years) and one from the northern and central regions of Jutland (Denmark, 50-70 years: PR=300/100,000; IR=58/100,000 person-years).”*

Re the sentence "important contribution of cardio-and cerebro-vascular, metabolic, neurological, and psychiatric diseases in explaining sex-differences in YOD incidence" in the conclusion: since dementia is a neurological disorder, please specify "other-than dementia neurological disorders"

🡺 Thank you very much, we modified the sentence accordingly.
