# Supplementary material for: Prevalence and incidence of young onset dementia and associations with comorbidities: A study of data from the French national health data system
Source: PLoS Med. 2021 Sep 23;18(9):e1003801. doi: 10.1371/journal.pmed.1003801 (PMC8496799; doi:10.1371/journal.pmed.1003801)
Supplement: Attachment — Submitted filename: Point by point response to reviewers and editor R2_VF.docx [file pmed.1003801.s007.docx]

**Point by point response to reviewers and editor’s comments**

Requests from Editors:

1. Title: Reviewer 3 makes a specific suggestion for the title; however, we suggest: “Prevalence and incidence of young-onset dementia and associations with comorbidities: A study of data from the French national health data system” or similar.

🡺 Thank you, we modified the title as suggested.

2. Data availability statement: The statement requires revision, as it currently reads that data are found in the supporting information files, which is not the case. Please include information explaining how individuals may request data access, such as: We suggest: “Data cannot be shared as access to the SNDS has to be approved by the French Data Protection Authority. Interested investigators can request their own access to the data according to the French law. Information on obtaining access to SNDS data can be found at <https://www.snds.gouv.fr/SNDS/Processus-d-acces-aux-donnees>. Requests for access to NSDS data must be submitted to the National Institute for Health Data (INDS)[[please provide a link or contact email]].”

🡺 We modified the data availability statement as suggested.

3. Throughout: Please replace "subject" with participant, patient, individual, or person.

🡺 We replaced the word “subject” by “individual” or “participant” throughout the text as appropriate.

4. Abstract: Methods and findings: Please include the number of individuals that are included among the 76% of the French population in the French National Health Data System, and please specify the name (Système national des données de santé (SNDS)). Please provide p values for the comparison of PR and IR between men and women. Please provide the result (OR with 95% CI and p value) for the association between specific comorbidities mentioned and incident YOD.

🡺 We added the number of individuals and p-values for the comparison of PR and OR between men and women. We also added age- and sex-adjusted p-values for the association between the comorbidities examined and incident YOD; however, it is not possible to add ORs and 95% IC as there are over 10 comorbidities (please see table 2).

5. Abstract: Conclusions: Line 45-46: Please revise to “...higher YOD frequency in men may be partly explained by higher prevalence…”

🡺 Thank you, we modified the wording.

6. Author summary: Why was this study done?: We suggest changing the second point to: “To the best of our knowledge, there is a lack of data regarding the frequency of young onset dementia…”

🡺 Done.

7. Author summary: What do these findings mean?: Please revise the final point to: “YOD incidence was higher in men than women, and this could possibly be due, at least in part, to the higher prevalence of…” or similar.

🡺 Done

8. In-text citations: Please place the reference bracket before the sentence punctuation, rather than after.

🡺 Done

9. Introduction: Line 74: We suggest noting here that young-onset dementia can also be referred to as early-onset dementia.

🡺 We added this precision (Introduction, line 78).

10. Introduction: Line 101: Here and throughout, please replace the term “subjects” with “individuals” or similar.

🡺 Done.

11. Methods: Ethical approval: Please state in the Methods that the relevant institutional review board(s) waived the need for ethical approval for the study.

🡺 As a governmental agency in charge of health surveillance in France, Santé publique France has a full and permanent access to SNDS data according to the French law to achieve it missions (French public health code, article R1461-14.1; https://www.legifrance.gouv.fr/codes/article_lc/LEGIARTI000037678676). This project is part of the surveillance program of neurodegenerative diseases in France and falls within the missions of Santé publique France. Therefore, there is no need for waiving ethical approval by an IRB. We added this precision in the manuscript line 129.

12. Methods: Analysis plan: Please make sure that the Methods section transparently describes when the described analyses were planned, and when/why any data-driven changes to analyses took place. Changes in the analysis-- including those made in response to peer review comments-- should be identified as such in the Methods section of the paper, with rationale. For example, please note whether the prevalence, incidence and comorbidities analyses were preplanned, and please note that the analysis of comorbidities in the context of sex differences was planned after observing differences in YOD between sexes. Please also note that the analysis of TBI was also added after the main analyses were completed.

🡺 We added a paragraph at the end of the statistical analysis section (line 184) to specify preplanned and changes in the analysis:

*« Analyses regarding prevalence, incidence, and associations of comorbidities with incident YOD were preplanned. As we found a strong difference in YOD incidence by sex, we further examined the contribution of comorbidities to sex-differences in YOD incidence by estimating the percentage reduction in the association between sex and YOD after adjustment for comorbidities. Traumatic brain injury was included after the main analyses were completed in response to a reviewer’s suggestion. »*

13. Methods: Line 111: We suggest “Data on hospitalizations in short stay departments....and at home are available.”

🡺 We modified the sentence accordingly (line 115).

14. Methods: Line 134, Line 153 and Line 159: Please replace “subjects” with “individuals” in these sentences.

🡺 Done.

15. Methods: Line 143-151: Please provide more details, or a list in the Supporting Information files, of the specific comorbidities assessed, unless this is described in supplementary table S1.

🡺 You are correct, the comorbidities are all listed in table S1.

16. Methods: Line 168-169: Please provide a reference to the National Institute of Statistics and Economic Studies data used here.

🡺 We added the following reference (line 176):

38.	Population en 2016. Recensement de la population - Base infracommunale (IRIS) [Internet]. 2016 [cited 2021 Aug 26]. Available from: <https://www.insee.fr/fr/statistiques/4228434>.

17. Methods: Line 180-181: Please include the reference to the STROBE Checklist in the Supporting Information file (S1 Checklist, or similar).

🡺 We now refer to S1 Strobe Checklist (line 190).

18. Results: Line 187-188: Please revise this sentence to clarify: “Women (12.7%) more often met all three criteria for inclusion compared to men (7.9%)...” or similar.

🡺 We modified the sentence as suggested (line 197):

“Women (12.7%) more often met all three criteria for inclusion compared to men (7.9%) (p<.0001).”

19. Results: Line 209: Please report p values as <0.001 where applicable.

🡺 Done (line 219).

20. Results: Line 219: Please clarify if “delusional disorders” is meant, or if psychotic disorder was intended.

🡺 As suggested in comments n°41 and 43, we replaced “delusional disorders” by “psychotic disorders” when appropriate throughout the manuscript and tables.

21. Discussion: Line 227: We suggest removing the word “robust” from the sentence.

🡺 We deleted the word “robust” and modified the first sentence of the first paragraph of the discussion so that it remains accurate after deleting this word (line 237):

*“We used data from the French national health data system for 76% of the French population to estimate the PR and IR of YOD in people aged 40 to 64 years-old.”*

22. Discussion: Line 231-232: We suggest revising to: “Rates of YOD were higher in men compared to women, and we identified that this association was attenuated by 55% after adjustment for all comorbid conditions considered, suggesting a potential role for comorbid conditions in the relationship between sex and YOD.” or similar.

🡺 We modified the sentence as suggested (line 242).

23. Discussion: Line 241: Please change to “...four catchment areas of eastern Sydney, Australia…”

🡺 We modified the sentence as suggested (line 253).

24. Discussion: Line 264: Please change “overestimate” to “overestimation” if accurate.

🡺 We modified the sentence as follows (line 280):

*« which may have led to an overestimation of the YOD frequency »*

25. Discussion: Line 267-270: Please revise this sentence to clarify, as the meaning is unclear: “A recent review on the accuracy of dementia diagnosis in routinely collected datasets reported good specificity and positive predictive value with moderate sensitivity,[24] comprised between 21%-86%, with only 3 of 12 studies reporting estimates ≥60%; however, sensitivity was higher at younger ages.[25, 26].”

🡺 We revised the sentence as follows and hope that is it clearer now (line 284):

*« A recent review on the accuracy of dementia diagnosis in routinely collected datasets reported good specificity and positive predictive value but moderate sensitivity[24]. Based on 12 studies, sensitivity was comprised between 21%-86%, with only 3 of 12 studies reporting estimates ≥60%. However, some studies found sensitivity to be higher at younger ages[25, 26]. »*

26. Discussion: Line 277: Please replace the term “subject” with “individuals” or similar.

🡺 Done.

27. Discussion: Line 313: Please clarify “inclusions of all persons <65 years” as your study seemed to consider adults less than 65 yrs of age but at least 40 years.

🡺 We modified the sentence as follows (line 331):

*« the inclusion of all persons aged 40 to 64 years-old affiliated to the general scheme of the French social security »*

28. Discussion: Line 322: Please replace “subject” with “individual” or similar.

🡺 Done.

29. Discussion: Line 324: Please replace “subject” with “individual” or similar. We suggest clarifying “overestimate” in this sentence by rephrasing “...which could be delayed relative to the actual age at incidence, leading to misclassification of some individuals with YOD as having LOD.” or similar.

🡺 We modified the sentence as follows (line 340):

*« In addition, we used the first date of contact for dementia with the medical system to define age at incidence which could be delayed relative to the actual age at incidence, leading to misclassification of some individuals with YOD as having LOD [59, 60]. »*

30. Discussion: Line 343: Limitations: Please comment on whether it is possible that people with Parkinson’s disease but not dementia are included in your study because they are receiving donepezil, for example?

🡺 In France, donepezil (Aricept) is not used to treat patients with Parkinson’s disease (PD) but without dementia. The only drug approved in France for PD patients with dementia is rivastigmine (Exelon), but again it is only used in PD patients who are demented.

31. Discussion: Line 344 and Line 348: Please replace the term “subject” with “individual” or similar.

🡺 Done.

32. Discussion: Line 360: We suggest revising to “Our results may suggest that preventive approaches targeting cardiovascular risk factors in mid-life, substance-abuse disorders, and prevention and managing traumatic brain injury could be further investigated as strategies for reducing or postponing young onset of dementia.” or similar, to make it clear that these results cannot demonstrate causal relationships between comorbidities and young onset dementia.

🡺 We modified the sentence as follows (line 379):

*« Our results may suggest that preventive approaches targeting cardiovascular risk factors in mid-life, substance-abuse disorders, and prevention and management of traumatic brain injury could be further investigated as strategies for reducing or postponing YOD. »*

33. Sections “Disclosure of potential conflicts of interest” “Funding” and “Availability of data and material” in the main manuscript text: Please remove these from the manuscript body, and please make sure all information is accurately entered with the manuscript submission metadata.

🡺 Done.

34. References: Please check the formatting of each reference in the list. Please use the "Vancouver" style for reference formatting, and see our website for other reference guidelines <https://journals.plos.org/plosmedicine/s/submission-guidelines#loc-references>

Specifically, please check the NLM abbreviations of journal titles; for example, “The Lancet Neurology” should be “Lancet Neurol” in references 1 and 2. Please also check capitalization (e.g. “Bmj” in reference 18).

🡺 Done.

35. Table 1 and Figure 1: Please define the abbreviation YOD in the legend.

🡺 Done.

36. Figure 1: Please clarify that the shaded region represents the 95% CIs.

🡺 Done.

37. Figure 2: Please clarify that the bars represent the 95% CIs. Please define IRR and PRR in the legend.

🡺 The legend of Figure 2 has been modified to be clearer:

« Male-to-female prevalence (PRR_M/W_) and incidence (IRR_M/W_) rate ratios, overall and by age. Black bars represent 95% confidence intervals (CI).”

38. Table 1: Please report p values as <.001 where relevant rather than <.0001.

🡺 Done.

39. Table 2 and Table 3: We suggest replacing “psychiatric diseases” with “disorders” and “delusional diseases” with “delusional disorders” in the tables.

🡺 Done. We also replaced “delusional disorders” by “psychotic disorders” when appropriate throughout the manuscript and tables (please see comments 41 and 43).

40. Table 2: In the footnote, please clarify the source of the comparison group “Overall French Population without YOD (2016). Please also report p values as <.001 where applicable.

🡺 Done.

41. Supplementary table 1: We suggest “psychiatric disorders” instead of “diseases” in the table. We suggest changing “delusional diseases” to “psychotic disorders” or similar, as the ICD-10 codes seem to include disorders related to psychosis, in addition to including delusional disorders. In the legend, please briefly explain “t” and “t-4” as used in the study.

🡺 Done.

42. Supplementary table 2: Please define YOD in the legend. Please clarify “(Rniam)” in the legend. Here and for supplementary table 3, please provide a reference for the number of persons/ person-years affiliated to the general scheme of the social security in 2016.

🡺 We clarified Rniam in supplementary tables 2 and 3 and added a link in supplementary table 2 to a file that shows the number of individuals affiliated to the general security scheme in 2016 (<https://assurance-maladie.ameli.fr/sites/default/files/2016_effectif-par-pathologie-classe-age-sexe_serie-annuelle.xls>). The number of person-years affiliated is not publicly available. *Santé Publique France* has an agreement with the *Caisse nationale d’Assurance Maladie* that provides these numbers each year to *Santé publique France*.

43. Supplementary table 4: We suggest “psychiatric disorders” instead of “diseases” in the table. We suggest changing “delusional diseases” to “psychotic disorders” or similar, as the ICD-10 codes seem to include disorders related to psychosis, in addition to including delusional disorders. Please also report p values as <.001 where applicable.

🡺 Done.

44. STROBE Checklist: We suggest renaming the file as S1 Checklist or S1 Strobe Checklist, or similar.

🡺 We renamed the file S1 Strobe Checklist.

**Comments from Reviewers:**

**Reviewer #1:** The authors have responded to each comment in turn.

🡺 Thank you again for your comments that helped to improve the final version of our manuscript.

Can the authors please re-visit the following conclusion, and change the tone from 'likely' to 'possibly' due to insufficient statistical power?

"Age-specific IRRM/W were similar to corresponding age-specific PRRM/W, and there was a similar trend of decreasing IRRM/W with continuous age but it was not statistically significant (p-interaction=0.494), likely due to insufficient statistical power as the number of incident cases in each age group was considerably smaller than the number of prevalent cases."

🡺 We modified the sentence as suggested (line 219).

**Reviewer #2:** The authors have sold all my comments addressing them clearly and correctly.

🡺 Thank you again for your comments that helped to improve the final version of our manuscript.

**Reviewer #3:** Thank you for sending a revision of this paper and for giving such a clear account of changes made. I have four minor points arising and have also listed a equally small number of spelling/grammatical changes to address.

🡺 Thank you again for your comments that helped to improve the final version of our manuscript.

1.	I noted the new title but would like to suggest streamlining to:

"Young onset dementia in France: prevalence, incidence and role of comorbidities."

🡺 The title was modified according as suggested by the editor: *« Prevalence and incidence of young-onset dementia and associations with comorbidities: A study of data from the French national health data system »*

2.	The editors asked that, in the abstract, you should quantify the main results (with 95% CIs and p values) - please check you have done this.

🡺 We added new precisions regarding missing p-value for sex differences in PRR and IRR.

3.	I also noted the request for you to include the (completed) STROBE checklist. I did not see the checklist in the supplementary materials, despite a heading on p.45.

🡺 We confirm that the completed Strobe checklist was uploaded with the revision. Is now named “S1 Strobe Checklist”.

4.	Lines 246-251. Can you hypothesise the reason for the lower rates in then Scottish, English and Japanese studies?

🡺 Thank you to have raised this point. We added a sentence to comment the discrepancy between our study results and those from earlier findings (line 263):

*“The inclusion of psychiatric hospitals records only in the Scottish study, the small catchment area and thus small number of incident cases in the English study, and a wide population as the denominator (18-64 years) in the Japanese study are the main explanations for the low incidence estimates in these studies.”*

Errors of spelling or grammar:

1.	Line 112: "After each hospital stay, a discharge summary is filled." - was this intended to say 'filed'?

2.	Line 234 - add two words: 'Previous Field studies have provided inconsistent results regarding YOD prevalence

3.	Line 264: which may have led to overestimation of YOD frequency.

4.	Lines 267-270 sentence beginning 'A recent review on the accuracy of dementia diagnosis …' would be better broken into two.

5.	Line 309: 'However, these findings ….'

🡺 Thank you very much for pointing out these errors. We have now made these corrections.

**Reviewer #4**: I have read the response to mine and to the other Reviews with great interest and appreciation. All my comments have been addressed and my questions answered properly. I strongly recommend publication.

🡺 Thank you again for your comments that helped to improve the final version of our manuscript.
